# Anomalous Intercoronary Communication with Unidirectional Flow in the Absence of Obstructive Coronary Artery Disease: A Case Report 

**Published:** 2016-10-03

**Authors:** Arash Gholoobi, Mohammad Vojdanparast

**Affiliations:** 1 *Atherosclerosis Prevention Research Center, Imam Reza Hospital, Mashhad University of Medical Sciences, Mashhad, Iran.*; 2 *Cardiovascular Research Center, Mashhad University of Medical Sciences, Mashhad, Iran.*

**Keywords:** *Coronary angiography*, *Coronary vessel anomalies*, *Congenital abnormalities*

## Abstract

Large intercoronary communications in the absence of obstructive coronary artery disease constitute a very rare coronary artery anomaly in which there is a readily visible connection between the 2 coronary arteries with a unidirectional or bidirectional blood flow; consequently, this anomaly may be misinterpreted as a functioning collateral vessel, indicative of an unrecognized proximal coronary artery occlusion. In contrast to collateral vessels that are seen in the presence of critical coronary artery stenosis and total occlusions, these arterial communications are vessels that are single, extramural, straight, and large in diameter. Myocardial ischemia could result from the coronary steal phenomenon by a unidirectional intercoronary communication. Herein, we describe a 57-year-old female with chest pain who was found in coronary angiography to have a single large intercoronary channel between the posterolateral branch of the right coronary artery and the distal left circumflex artery with a unidirectional flow.

## Introduction

In the normal coronary circulation, although there are some intercoronary and intracoronary anastomotic channels, they are functionally insignificant and are too small to be visualized by conventional coronary angiography. However, the development of a severe coronary stenosis or total occlusion increases the flow through these channels and hence their size, making them readily visible on coronary angiography. Large intercoronary communications in the absence of obstructive coronary artery disease constitute a very rare coronary artery anomaly.^1^^, ^^2^ Herein, we describe a patient with this type of anomaly found incidentally on coronary angiography.

## Case Report

A 57-year-old female with chest pain and palpitation as chief symptoms was referred to us for coronary angiography. She had been discharged from another hospital with a diagnosis of probable acute coronary syndrome. She only had a history of hypertension. Laboratory findings were unremarkable with normal fasting plasma glucose and lipid profile and negative cardiac enzymes. Electrocardiogram (ECG) was normal except for frequent premature atrial contractions. Echocardiography also revealed normal left ventricular systolic function with mild hypertrophy and no regional wall motion abnormality. There was grade 1 diastolic dysfunction.

**Figure 1:**
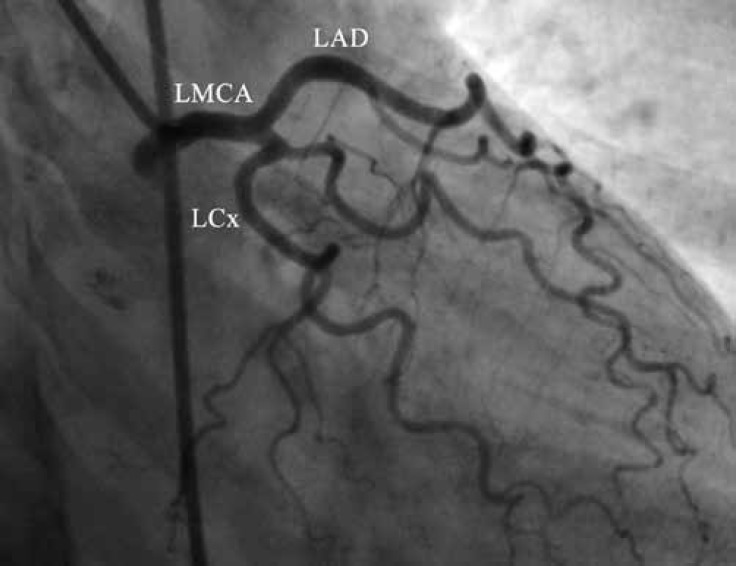
Coronary angiography in the right anterior oblique projection with caudal angulation shows a normal left coronary system

LAD, Left anterior descending artery; LCx, Left circumflex artery; LMCA, Left main coronary artery

**Figure 2:**
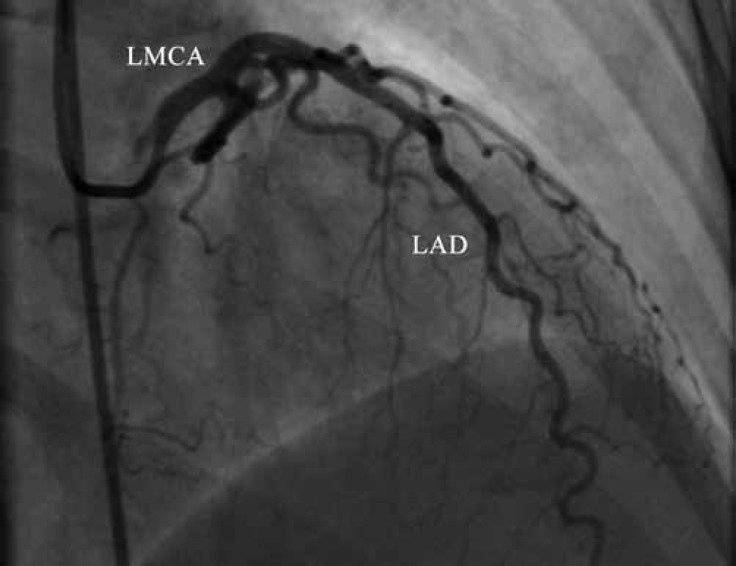
Coronary angiography in the right anterior oblique projection with cranial angulation shows a normal left coronary system.

LAD, Left anterior descending artery; LMCA, Left main coronary artery

**Figure 3:**
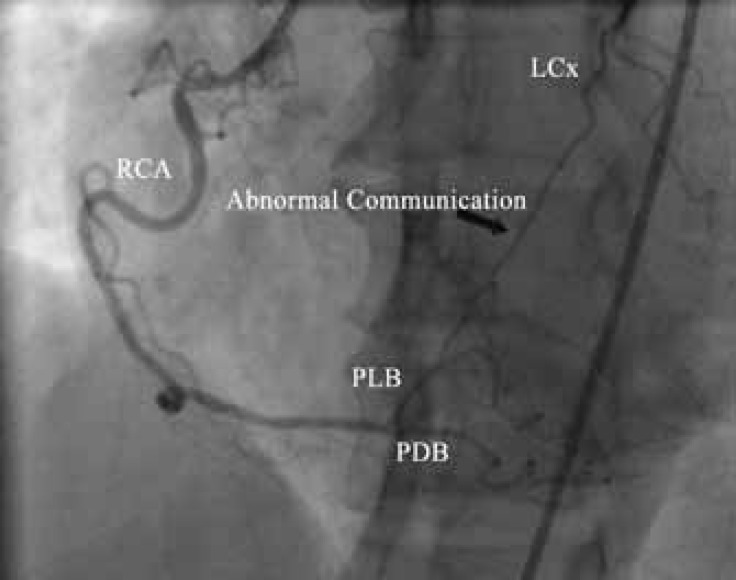
Coronary angiography in the straight left anterior oblique projection reveals a normal right coronary artery. There is a single large channel (black arrow) connecting the posterolateral branch to the distal left circumflex artery.

LCx, Left circumflex artery; RCA, Right coronary artery; PDB, Posterior descending branch; PLB, Posterolateral branch

**Figure 4:**
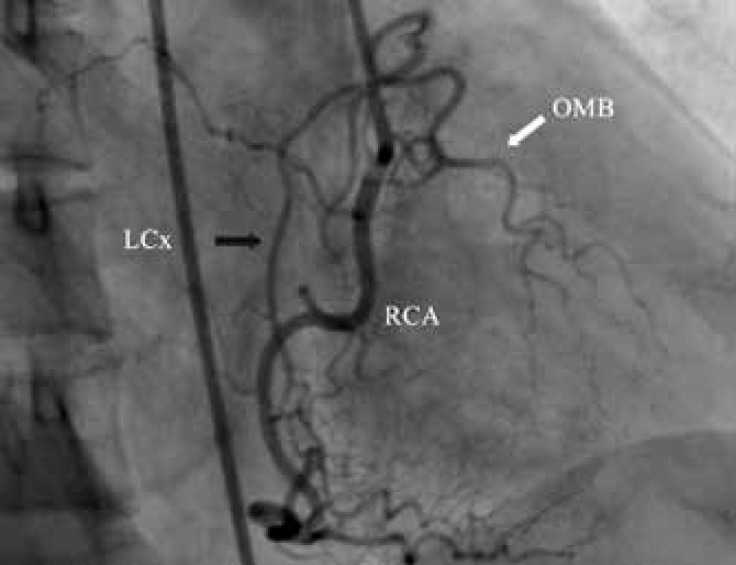
Coronary angiography in the straight right anterior oblique projection reveals a normal right coronary artery. There is a single large channel (black arrow) connecting the posterolateral branch to the distal left circumflex artery, which opacifies the obtuse marginal branch (white arrow) as well.

LCX, Left circumflex artery; OMB, Obtuse marginal branch; RCA, Right coronary artery

Coronary angiography showed a normal left coronary system (Figure 1 and Figure 2.). During right coronary artery (RCA) injection, a single abnormal vessel was seen connecting the posterolateral branch to the distal left circumflex (LCx) artery and opacifying the distal LCx as well as the obtuse marginal branch in a retrograde fashion (Figure 3 and Figure 4.).

## Discussion

Anomalous intercoronary communications constitute a very rare coronary artery anomaly. In contrast to collateral vessels that are seen in the presence of critical coronary artery stenosis and total occlusions, these arterial communications are vessels that are single, extramural, straight, and large in diameter-as was the case in our patient. The histological structure of these single channels has the characteristics of a normal arterial wall, with a well-defined muscular layer.^3^^, ^^4^

In the largest study performed to date by Yamanaka et al. on 126,595 patients undergoing coronary angiography, only 3 (0.002%) patients with this type of anomaly were reported.^1^ The intercoronary connection was between the atrioventricular branch of the RCA and LCx arteries with bidirectional flows so that the contrast injection of the RCA filled the LCx artery in a retrograde fashion and the left coronary system injection opacified the RCA retrogradely as well. Furthermore, another communication was reported between the distal left anterior descending (LAD) artery and distal posterior descending (PD) branch of the RCA. The communications were found in the absence of coronary atherosclerosis. 

Paolo and colleagues reported an intercoronary communication with a bidirectional flow between the right PD branch and the distal LCx artery in a 56-year-old man with no coronary atherosclerosis.^2^

Soo Hyun et al. reported the incidental finding of an intercoronary communication between the LCx and the RCA with a bidirectional flow in a patient with coronary vasospastic angina in the LAD artery.^3^ They concluded that the provocative test for coronary vasospasm could be considered if an intercoronary communication was found in patients with chest pain and no obstructive coronary artery disease, albeit the relation between intercoronary connections and coronary artery spasm remains unproved. 

Some authors believe that these connections may play a protective role for the myocardium, if the coronary artery obstruction develops in 1 of the 2 connecting vessels.^2^^-^^5^ On the other hand, myocardial ischemia could be the result of the coronary steal phenomenon by an unidirectional intercoronary communication.^3^^, ^^4^^, ^^6^ Sengül et al. demonstrated the evidence of ischemia by myocardial perfusion scintigraphy in a 58-year-old man with an arterial connection between the right PL branch and the distal LCx artery with a unidirectional flow in the presence of otherwise normal coronary arteries-as was the case in our patient.^6^ However, we do not have any proof of objective myocardial ischemia in our patient, and nor do we have evidence that her chest pain could be noncardiac in origin. Linsenmeyer and Schneider reported a 57-year-old woman with typical angina who was found to have an intercoronary communication between the right PD branch and the distal LAD artery with a unidirectional flow in a manner whereby the RCA injection filled the LAD artery retrogradely up to the mid portion but the right PD branch was not visualized during the left coronary system injection.^7^ Nonetheless, there was no evidence of myocardial ischemia by exercise myocardial perfusion scintigraphy in this patient without obstructive coronary disease. Furthermore, this unidirectional flow may be due to the lower velocity of the flow in the left coronary artery because of its larger vascular capacity. Super selective LCx or LAD artery injection might aid to prove the true one-way functionality of these communications.^5^ Nevertheless, neither we nor previous studies have performed this modality. All together, the ischemic potential of these communications with unidirectional flows remains controversial. 

The absence of obstructive coronary artery disease along with similar morphological features and the 2 distinct locations in all the reported cases supports the congenital nature of this anomaly.^1^^-^^7^ It is worth noting that the intercoronary connection between the RCA and the LCx artery could be a variant of what is originally termed “Kugel’s collateral”.^8^

## Conclusion

Anomalous intercoronary communications comprise a very rare coronary artery anomaly where there is a single, straight, and large arterial communication between the distal branches of RCA (PD or PL branches) and the distal LAD or LCx with unidirectional or bidirectional blood flows. One should be aware of this type of coronary anomaly so as not to misinterpret it as a functioning collateral vessel indicative of an unrecognized proximal coronary artery occlusion.

## Notes:

***This paper should be cited as: ***Gholoobi A, Vojdanparast M. Anomalous Intercoronary Communication with Unidirectional Flow in the Absence of Obstructive Coronary Artery Disease: A Case Report. J Teh Univ Heart Ctr 2016;11(4):203-205.
